# Pulmonary fungus ball caused by *Penicillium capsulatum* in a patient with type 2 diabetes: a case report

**DOI:** 10.1186/1471-2334-13-496

**Published:** 2013-10-23

**Authors:** Min Chen, Jos Houbraken, Weihua Pan, Chao Zhang, Hao Peng, Lihui Wu, Deqiang Xu, Yiping Xiao, Zhilong Wang, Wanqing Liao

**Affiliations:** 1 Shanghai Key Laboratory of Molecular Medical Mycology, Department of Dermatology, Shanghai Changzheng Hospital, Shanghai, China; 2 Department of Applied and Industrial Mycology, CBS-KNAW Fungal Biodiversity Centre, Utrecht, the Netherlands; 3 Department of Thoracic Surgery, Shanghai Changzheng Hospital, Shanghai, China; 4 The College of Life Science, Fudan University, Shanghai, China

**Keywords:** Fungal ball, Pulmonary infections, Penicillium capsulatum

## Abstract

**Background:**

Following the recent transfer of all accepted species of *Penicillium* subgenus *Biverticillium* to *Talaromyces* (including *Talaromyces marneffei*, formerly *Penicillium marneffei*), *Penicillium* species are becoming increasingly rare causal agents of invasive infections. Herein, we present a report of a type 2 diabetes patient with a fungus ball in the respiratory tract caused by *Penicillium capsulatum*.

**Case presentation:**

A 56-year-old Chinese female gardener with a 5-year history of type 2 diabetes presented at the Shanghai Changzheng Hospital with fever, a cough producing yellow-white sputum, and fatigue. The therapeutic effect of cefoxitin was poor. An HIV test was negative, but the β-D-glucan test was positive (459.3 pg/ml). Chest radiography revealed a cavitary lesion in the left upper lobe, and a CT scan showed globate cavities with a radiopaque, gravity-dependent ball. The histopathologic features of the tissue after haematoxylin-eosin staining showed septate hyphae. The fungus was isolated from the gravity-dependent ball and identified as *Penicillium capsulatum* based on the morphological analysis of microscopic and macroscopic features and on ribosomal internal transcribed spacer sequencing. After surgery, the patient was cured with a sequential treatment of fluconazole 400 mg per day for 90 days and caspofungin 70 mg per day for 14 days.

**Conclusions:**

Although the prognosis is often satisfactory, clinicians, mycologists and epidemiologists should be aware of the possibility of infection by this uncommon fungal pathogen in diabetes patients, since it may cause severe invasive infections in immunocompromised hosts such as diabetes and AIDS patients.

## Background

Penicilliosis is an invasive fungal infection that primarily occurs in the southeast and eastern regions of Asia, including Thailand, northeast India, China, Hong Kong, Laos, Cambodia, Malaysia, Myanmar, Vietnam and Taiwan [1]. *Penicillium marneffei* causes the vast majority of penicillioses, especially in immunosuppressed hosts such as AIDS patients and otherwise healthy individuals [1-3]. This species was recently transferred to the genus *Talaromyces* together with other *Penicillium* species belonging to the subgenus *Biverticillium*[4]. Thus, excluding *Talaromyces marneffei* (formerly known as *P. marneffei*), systemic infections caused by *Penicillium* species are becoming increasingly rare. Currently, approximately 300 species are grouped within the genus *Penicillium*, and only a limited number are associated with invasive fungal infections [5]. The majority of *Penicillium* species are saprobic and commonly occur in soil; however, some species are known for their positive or negative effects on humans. The positive impacts include their use in food fermentation and the production of drugs, and the negative effects are related to the production of mycotoxins, the induction of hypersensitivity reactions (e.g., asthma and extrinsic allergic alveolitis) and the infection of humans. Presently, China has an increasing burden of diabetes (92.4 million adults above the age of 20) [6], and diabetes mellitus is considered to be a significant suppressor of the human immune system. Herein, we report a case of a 56-year-old patient with type 2 diabetes who developed a fungus ball in the left lung caused by *Penicillium capsulatum*. The diagnosis, treatment and clinical importance of *P. capsulatum* are discussed.

## Case presentation

**Figure 1:**
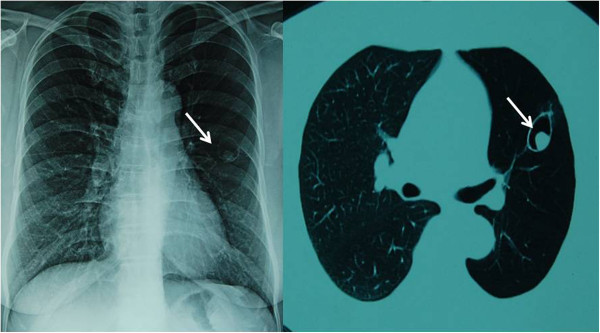
Chest computed tomography showing a fungus ball in the left upper lobe of the lung.

**Figure 2:**
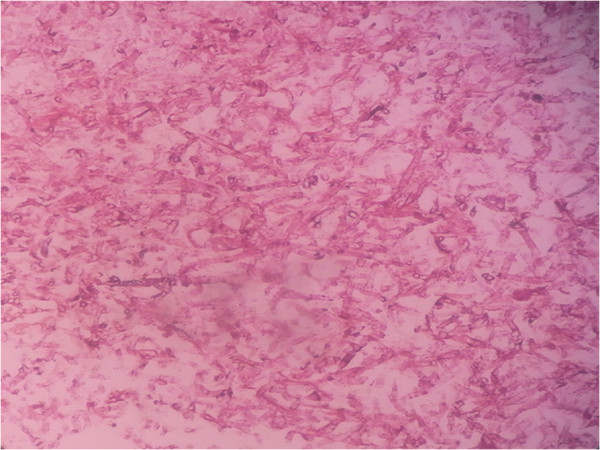
Histologic section of a specimen taken from the fungus ball showing septate fungal hyphae (H&E strain) (magnification × 400).

**Figure 3:**
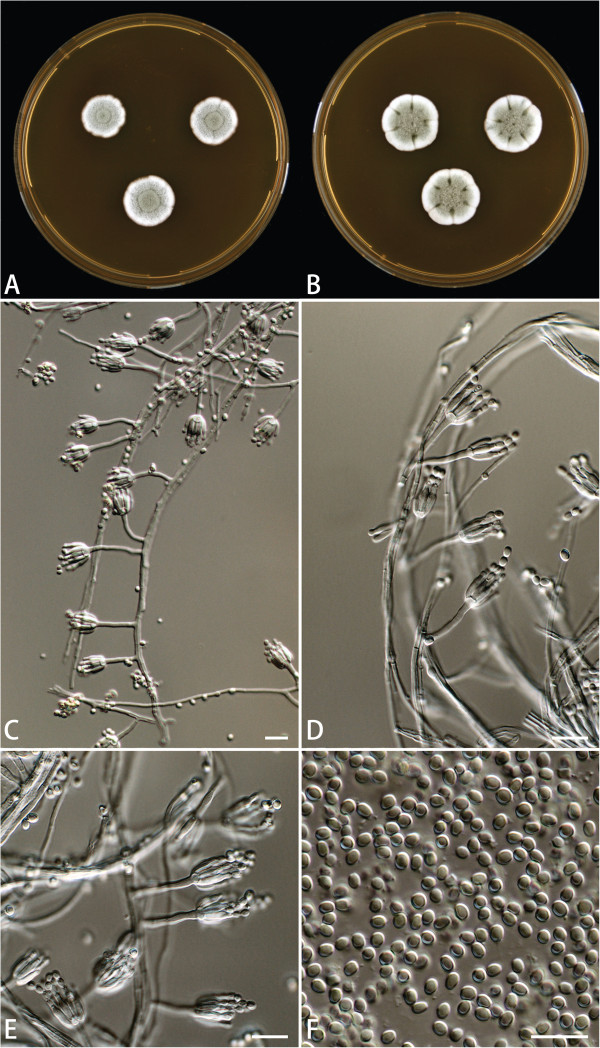
***Penicillium capsulatum *CBS 134186. A**. 7 days old culture on MEA incubated at 25°C. **B**. 7 days old culture on MEA incubated at 37°C. **C**–**E**. Conidiophores. **F**. Conidia. Scale bars = 10 μm.

**Figure 4:**
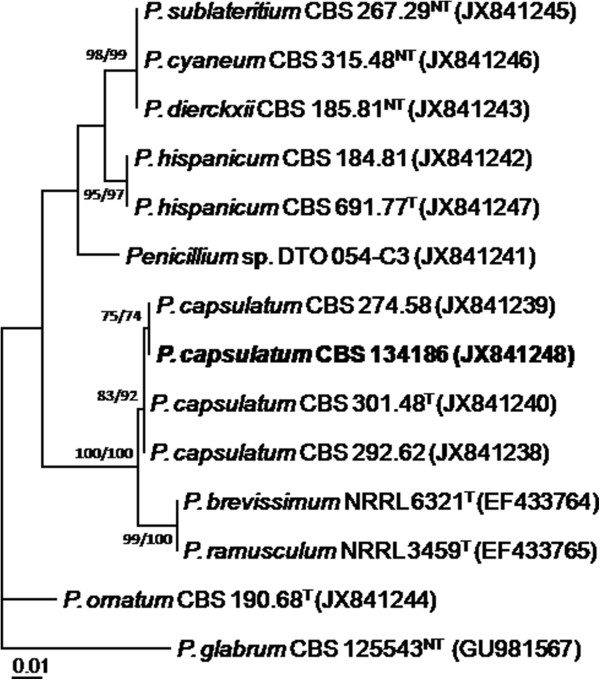
**The best-scoring Maximum Likelihood tree with the highest log likelihood (−1269.6815) is presented and shows that the strain isolated from gravity-dependent ball (in bold) is similar to the type of *P. capsulatum *CBS 301.48.** The phylogram was constructed using MEGA5.1 and is based on ITS sequences. The bootstrap percentages using Maximum Parsimony (MP) and Maximum Likelihood (ML) (MP/ML) are shown at the nodes. Values less than 70% support are not shown; the tree is rooted with *Penicillium glabrum* CBS 125543 (T = Type; NT = Neotype).

A 56-year-old Chinese female gardener presented to the Shanghai Changzheng Hospital with fever, a cough producing yellow-white sputum and fatigue. The therapeutic effect of cefoxitin (4000 mg/day, 14 days) was poor. She had a 5-year history of type 2 diabetes with effective treatment by insulin injection but no history of tuberculosis, chronic obstructive pulmonary disease, asthma or other underlying immunosuppressive diseases. A test for the human immunodeficiency virus was negative, but the β-D-glucan test was positive (459.3 pg/ml). Chest radiography revealed a cavitary lesion in the left upper lobe, and chest computed tomography (CT) showed globate cavities with a radiopaque, gravity-dependent ball (Figure 1). Based on these data, the causal agent of the infection was initially thought to be *Aspergillus* spp. because of the fungus ball’s resemblance to an aspergilloma caused by species such as *Aspergillus fumigatus*. Histologic sections of the tissue were stained with haematoxylin and eosin (HE) and showed septate fungal hyphae (Figure 2). Specimens from the gravity-dependent ball were obtained, and the fungal strain was identified as *Penicillium capsulatum* via micro- and macro-morphology examination and molecular sequence analysis. For macroscopic examination, the culture was grown for 7 days at 25°C on malt extract agar (MEA), Czapek yeast extract agar (CYA) and yeast extract sucrose agar (YES), and the fungus exhibited restricted growth on all media (13–17 mm) with grey-green sporulating colonies. Growth on MEA plates incubated at 30°C and 37°C was faster (17–25 mm) than at 25°C. Microscopy analysis of the colonies grown on MEA showed short, smooth-walled, monoverticillate *Penicillium* conidiophores. The conidia were smooth walled and ellipsoid to slightly cylindrical and measured 3.5–4.5 × 2.5–3.0 μm (Figure 3). Molecular characterisation of the strain was performed by PCR amplification and sequencing of the ribosomal internal transcribed spacer region as described by Houbraken *et al*. [7]. The isolate was deposited in the CBS collection under the accession number CBS 134186, and newly generated sequences were deposited in GenBank. Figure 4 shows that the strain belongs to *Penicillium* section *Ramigena*[5] and that it is closely related to the type culture of *Penicillium capsulatum*, CBS 301.48. So we diagnosed this infection as Penicillioma which we created particularly. After pulmonary lobectomy, the patient was cured sequentially by treatment of fluconazole (400 mg/day, 90 days) and caspofungin (70 mg/day, 14 days).

Furthermore, to confirm the antifugal susceptibility in vitro, the clinical isolate CBS 134186 was, together with other environmental *P. capsulatum* isolates, tested in vitro against seven antifungal agents (amphotericin B, voriconazole, itraconazole, ketoconazole, fluconazole, 5-fluorocytosine, and caspofungin) using the CLSI M38-A2 broth microdilution method [8]. The results of this analysis are summarized in Table 1 and show that all tested *P. capsulatum* strains have a low minimal effective concentration (MEC) to caspofungin and a high minimal inhibitory concentration (MIC) to fluconazole. No substantial difference for all seven antifungal agents in antifungal susceptibility was observed between CBS 134186 and the *P. capsulatum* strains isolated from environmental sources (Table 1).

**Table 1:** **Antifungal susceptibilities of *P. capsulatum *** **isolates from strains isolated from environmental sources and CBS 134186 (μg/mL)**

	**Amphotericin B**	**Voriconazole**	**Itraconazole**	**Ketoconazole**	**Fluconazole**	**5-fluorocytosine**	**Caspofungin**
ATCC10420	1	1	0.25	0.125	>64	16	1
ATCC48735	1	0.5	0.25	0.0625	>64	32	1
CBS134186	0.5	0.5	0.25	0.0625	>64	32	1

## Conclusions

As reviewed by Lyratzopoulos *et al*. [9], only fifteen cases of invasive infections caused by species other than *T. marneffei* have been linked to the *Penicillium* genus worldwide. More recently, other *Penicillium* species, such as *P. chrysogenum*, *P. piceum* and *P. purpurogenum*, were found to be associated with these infections [10-13]. However, when applying the current taxonomic concepts [4], the latter two *Penicillium* species now belong to the *Talaromyces* genus, namely *T. purpurogenus* and *T. piceus*.

*Penicillium capsulatum*, a rare species belonging to *Penicillium* section *Ramigena*[5], is considered a useful microorganism in the paper manufacturing industry [14,15], but it has never been previously recognised as a causative agent of invasive infection in humans. One of the pathogenicity factors of *Penicillium* species causing invasive infections is its ability to grow at 37°C [16]. The majority of *Penicillium* species have a maximum growth temperature below 37°C; however, exceptions include *P. chrysogenum*, *P. citrinum*, *P. decumbens* and *P. janthinellum*, and some of these species are causal agents of invasive infections, as stated above [10,16]. Our clinical *P. capsulatum* isolate grows well on MEA, CYA and YES incubated at 37°C, and growth at 30°C and 37°C was faster (17–25 mm) than at 25°C (13–17 mm). In our opinion, both the fungus’s ability to grow at 37°C and the patient’s diabetes mellitus contributed to the presence of the fungus ball in the patient. Despite the clinical strain can be considered resistant to fluconazole, the treatment seemed to be effective in vivo in this case. We believe that combined antifungal drug (caspofungin + fluconazole), resection of the primary lesion, and the patient’s immune status are important for satisfactory prognosis. Additionally, there is no substantial difference in antifungal susceptibilities between the *P. capsulatum* strains isolated from environmental sources and the CBS 134186 isolate (Additional file 1: Table S1).

In the present study, we used phenotypic characters and ITS sequences for identification. Identification based solely on the phenotype is often difficult and requires well-trained staff. Currently, molecular-based techniques, especially DNA sequencing, are frequently used for identification, and the ITS region is accepted as the primary fungal barcode [17]. This study shows that *P. capsulatum* can be identified based on ITS sequence data alone. In contrast, other *Penicillium* (and *Aspergillus*) species cannot be unambiguously identified by ITS sequencing alone, and partial β-tubulin or calmodulin sequences are required to ensure correct species identification [7]. The initial results based on the chest radiography and CT scan showed structures resembling an aspergilloma caused by *Aspergillus*. *Penicillium* and *Aspergillus* are sister genera and belong to the family *Aspergillaceae*[12]. This close relationship may explain their resemblance in chest radiographs and CT scans. It should be noted that *Aspergillus* species cause most pulmonary mycetomas, and other phylogenetically unrelated fungi including *Fusarium*, *Mucor* and *Paecilomyces*, can cause similar clinical manifestations [18].

In summary, *Penicillium capsulatum* produced a pulmonary fungus ball in a female patient with type 2 diabetes but was cured with combined surgical and antifungal treatment. An aggressive diagnostic and therapeutic strategy should be pursued for such infections. To our knowledge, this is the first report of a pulmonary infection caused by *P. capsulatum* worldwide; thus, this report expands our understanding of the pathogenicity of the genus *Penicillium* in this age of global warming [19]. Clinicians, mycologists and epidemiologists should be aware of the possibility of infection by uncommon fungal pathogens in patients because emerging pathogenic fungi are increasingly recognised as major threats to human health [19,20].

## Consent

Written informed consent was obtained from the patient for publication of this Case report and any accompanying images. A copy of the written consent is available for review by the Series Editor of this journal.

## Competing interests

The authors declare that they have no competing interests.

## Authors’ contributions

MC collected the clinical data, performed part of molecular identification and drafted the manuscript. JH performed the phenotypic and molecular identification and helped with the draft of the manuscript. WP carried out the histologic examinations. CZ performed the susceptibility test. HP collected the clinical specimen and the clinical data. LW and ZW collected the clinical specimen. DX and YX performed the phenotypic identification. WL supervised the clinical case interpretation, participated in the coordination and concept of the manuscript, and helped with the draft of the manuscript. All authors have read and approved the manuscript.

## Pre-publication history

The pre-publication history for this paper can be accessed here:

http://www.biomedcentral.com/1471-2334/13/496/prepub

## Supplementary Material

Additional file 1: Table S1 Antifungal susceptibilities of *P. capsulatum* isolates from environmental or clinical sources (μg/mL). Click here for file
