# Supplementary material for: Pulmonary fungus ball caused by Penicillium capsulatum in a patient with type 2 diabetes: a case report
Source: BMC Infect Dis. 2013 Oct 23;13:496. doi: 10.1186/1471-2334-13-496 (PMC3819729; doi:10.1186/1471-2334-13-496)
Supplement: Additional file 1: Table S1 — Antifungal susceptibilities of P. capsulatum isolates from environmental or clinical sources (μg/mL). [file 1471-2334-13-496-S1.docx]

Table S1. Antifungal susceptibilities of *P. capsulatum* isolates from environmental or clinical sources (µg/mL)

|  | amphotericin B | voriconazole | itraconazole | ketoconazole | fluconazole | 5-fluorocytosine | caspofungin |
| --- | --- | --- | --- | --- | --- | --- | --- |
| ATCC10420 | 1 | 1 | 0.25 | 0.125 | >64 | 16 | 1 |
| ATCC48735 | 1 | 0.5 | 0.25 | 0.0625 | >64 | 32 | 1 |
| CBS134186 | 0.5 | 0.5 | 0.25 | 0.0625 | >64 | 32 | 1 |
